# Microbiome of lovebug (*Plecia longiforceps*) in Seoul, South Korea

**DOI:** 10.1128/spectrum.03809-23

**Published:** 2024-05-29

**Authors:** Myung-hee Yi, Jun Ho Choi, Myungjun Kim, Xavier Chavarria, Sohyeon Yun, Singeun Oh, Dongjun Kang, Tai-Soon Yong, Ju Yeong Kim

**Affiliations:** 1 Department of Tropical Medicine, Institute of Tropical Medicine, and Arthropods of Medical Importance Resource Bank, Yonsei University College of Medicine, Seoul, South Korea; Fujian Agriculture and Forestry University, Fuzhou City, China

**Keywords:** lovebug, *Plecia longiforceps*, microbiome, *Rickettsia*, *Pandoraea*

## Abstract

Lovebugs appeared in large numbers across a wide area in Seoul, South Korea, in June 2023. The sudden appearance of exotic insects not only discomforts people but also fosters anxiety, as their potential for pathogen transmission would be unknown. In this study, targeted next-generation sequencing (NGS) of the 16S rRNA gene V4 region was performed using iSeq 100 to screen for bacteria in lovebugs. Forty-one lovebugs (20 females and 21 males) collected in Seoul, Korea, were identified as *Plecia longiforceps* based on mitochondrial cytochrome oxidase subunit 1 sequencing data using PCR. We analyzed the microbiome of the lovebugs and detected 453 species of bacteria. Among all bacteria screened based on NGS, *Rickettsia* was detected in all samples with an average relative abundance of 80.40%, followed by *Pandoraea* and *Ewingella*. Diversity (alpha and beta) between females and males did not differ; however, only *Tumebacillus* showed a higher relative abundance in females. Sequencing analysis of *Rickettsia* using a *gltA* gene-specific primer by PCR showed that it had higher sequence similarity to the *Rickettsia* symbiont of arthropods than to the spotted fever group rickettsiae. Eleven samples in which *Pandoraea* was detected by iSeq 100 were confirmed by PCR and exhibited 100% sequence identity to *Pandoraea oxalativorans* strain DSM 23570. Consequently, the likelihood of pathogen transmission to humans is low. The applied method may play a crucial role in swiftly identifying bacterial species in the event of future outbreaks of exotic insects that may be harmful to humans.

**IMPORTANCE:**

Lovebugs have recently emerged in large numbers in Seoul, causing major concern regarding potential health risks. By performing the next-generation sequencing of the 16S rRNA gene V4 region, we comprehensively examined the microbiome of these insects. We identified the presence of numerous bacteria, including *Rickettsia* and *Pandoraea*. Reassuringly, subsequent tests confirmed that these detected bacteria were not pathogenic. The present study addresses health concerns related to lovebugs and shows the accuracy and efficiency of our detection technique. Such methods prove invaluable for rapidly identifying bacterial species during potential outbreaks of unfamiliar insects, thereby ensuring public safety.

## INTRODUCTION

The lovebug inhabits subtropical East Asia, including southeastern China, Taiwan, and Japan’s Ryukyu Islands (1–3). In South Korea, a lovebug outbreak was reported for the first time in 2022 (4). After an unprecedented outbreak was reported in northwestern Seoul in 2022, lovebugs spread to southern Seoul in the summer of 2023, causing concern among residents and health authorities. Lovebugs have been found on residential streets, building walls, and glass windows; however, by 2023, they were also found on Seoul mountaintops, presumably expanding their habitat (5). Recently, two lovebug species, *Plecia longiforceps* and *Plecia thulinigra*, were identified in various regions of Korea (4, 6).

An outbreak of *Plecia nearctica* Hardy (Diptera: Bibionidae) has been previously reported in Florida, USA (7). Lovebugs are non-toxic and harmless but are a nuisance to people, as they tend to cling to clothing and vehicles after invading residential areas and local shops (8). In particular, *P. nearctica* is considered a nuisance pest because it potentially adversely affects beekeeping due to hostile interactions with honeybees, and its acidic fluids corrode clear coatings on cars (9, 10).

Although Korea has experienced massive lovebug outbreaks, research on the bacteria they carry, especially those that are potentially pathogenic to humans, remains limited. Therefore, it is crucial to investigate and understand potential risks. In this study, to determine whether lovebugs could cause human health problems, we performed bacterial metabarcoding using iSeq 100 to screen for pathogens in *P. longiforceps* and confirmed their presence by PCR using specific primers.

## RESULTS

### Identification of lovebug species

Based on the mitochondrial cytochrome oxidase subunit 1 (COI) sequencing data, all specimens in this study were 99.42% identical to *P. longiforceps* sequences previously investigated in Korea. All samples had the same COI sequence, indicating that they were established from a single invasion.

### Lovebug microbiome composition

**Fig 1:**
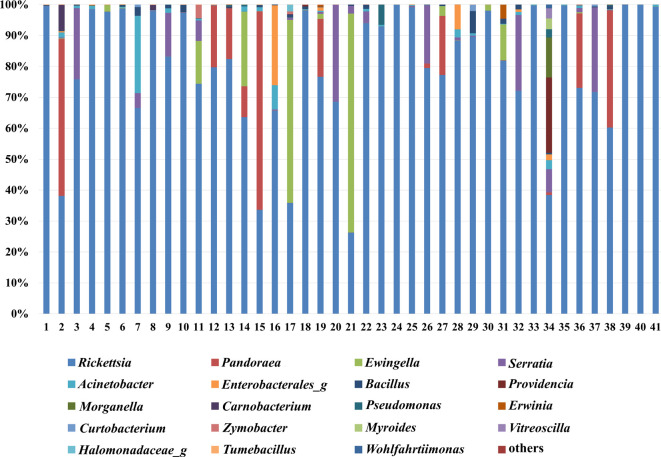
Microbiome composition of each lovebug at the genus level (*n* = 41). Reads accounting for more than 0.1% of total reads are shown.

The high-throughput sequencing of the 16S rRNA gene of 41 *P*. *longiforceps* lovebug samples using iSeq 100 produced an average (±SD) total read count of 25,302 ± 8,873. We analyzed the lovebug microbiome and detected 284 bacterial genera using the EzBio-Cloud database. The relative abundance of bacterial taxa in the microbiomes of individual lovebugs is shown in Fig. 1. At the species level, all samples were dominated by *Rickettsia* (26.19–99.86% of the total community, average 80.40%) (Fig. 1). Bacterial genera, such as *Pandoraea*, *Ewingella*, *Serratia*, and others, were detected in some lovebug samples, but only *Rickettsia* was detected in all 41 samples. The second most abundant bacterium, *Pandoraea*, was detected in 11 of the 41 lovebug samples [6 out of 20 females (30.00%) and 5 out of 21 males (23.81%)]. Additionally, the results of the analysis of the relative abundance of bacterial taxa using QIIME 2 based on SLIVA 138.1 data were confirmed to be no different from the results of the analysis using the EzBio-Cloud database (Fig. S1).

### Differences in bacterial composition between female and male lovebugs

**Fig 2:**
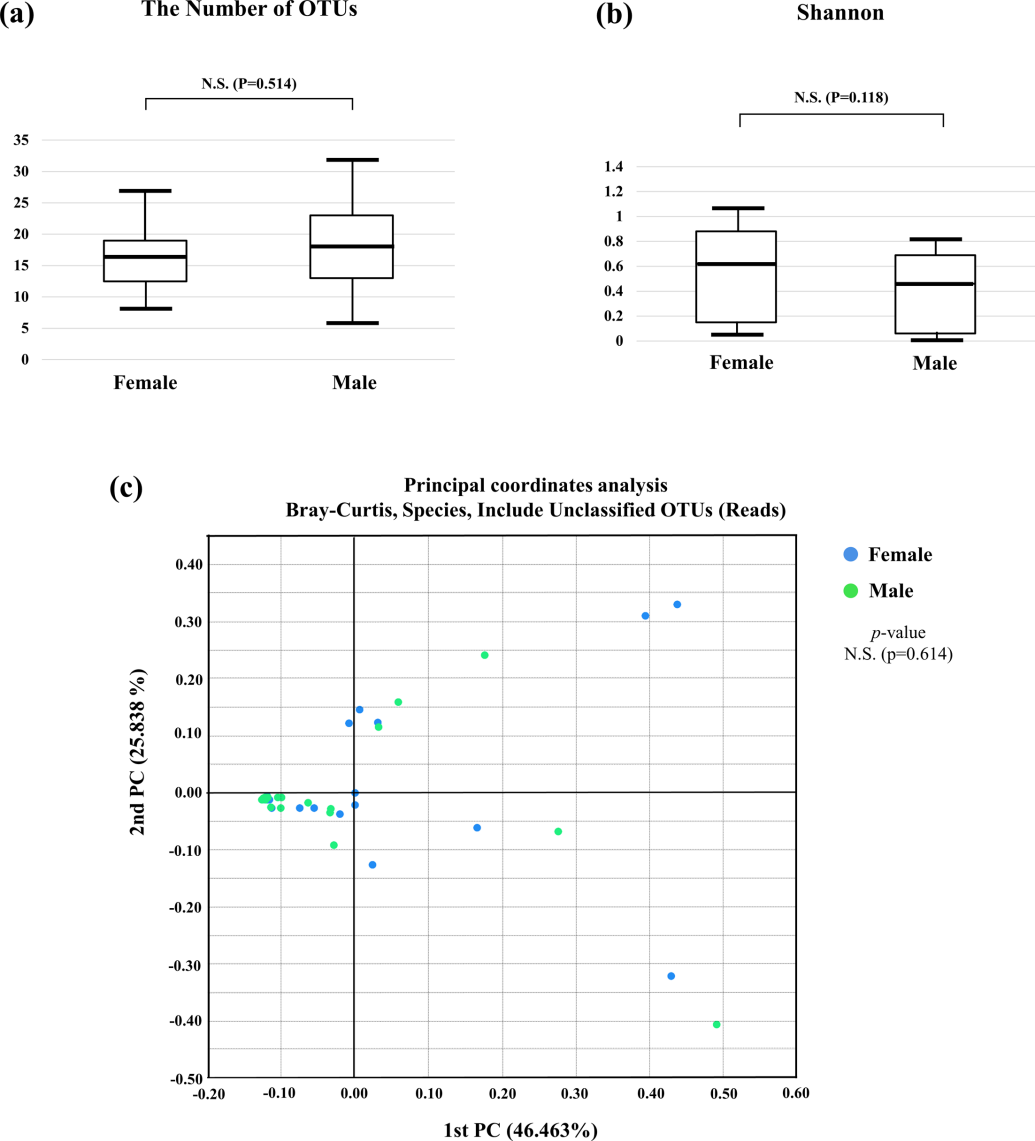
Alpha and beta diversities of female and male lovebug microbiomes. (**a**) The number of operational taxonomic units and (**b**) Shannon index between the female (*n* = 20) and male (*n* = 21) groups. (**c**) Principal coordinate analysis of the microbiome composition of female and male lovebugs.

We investigated the differences in bacterial composition between female and male lovebugs. The number of operational taxonomic units (OTUs) did not significantly differ between female and male lovebugs (Fig. 2a). In addition, the Shannon index (bacterial diversity) did not differ significantly between the two groups (Fig. 2b). The principal coordinate analysis (PCoA) results (Fig. 2c) demonstrated that the samples were not clustered based on sex (*P* = 0.614 in PERMANOVA), suggesting that the bacterial composition of the lovebugs did not differ significantly between female and male lovebugs.

**Fig 3:**
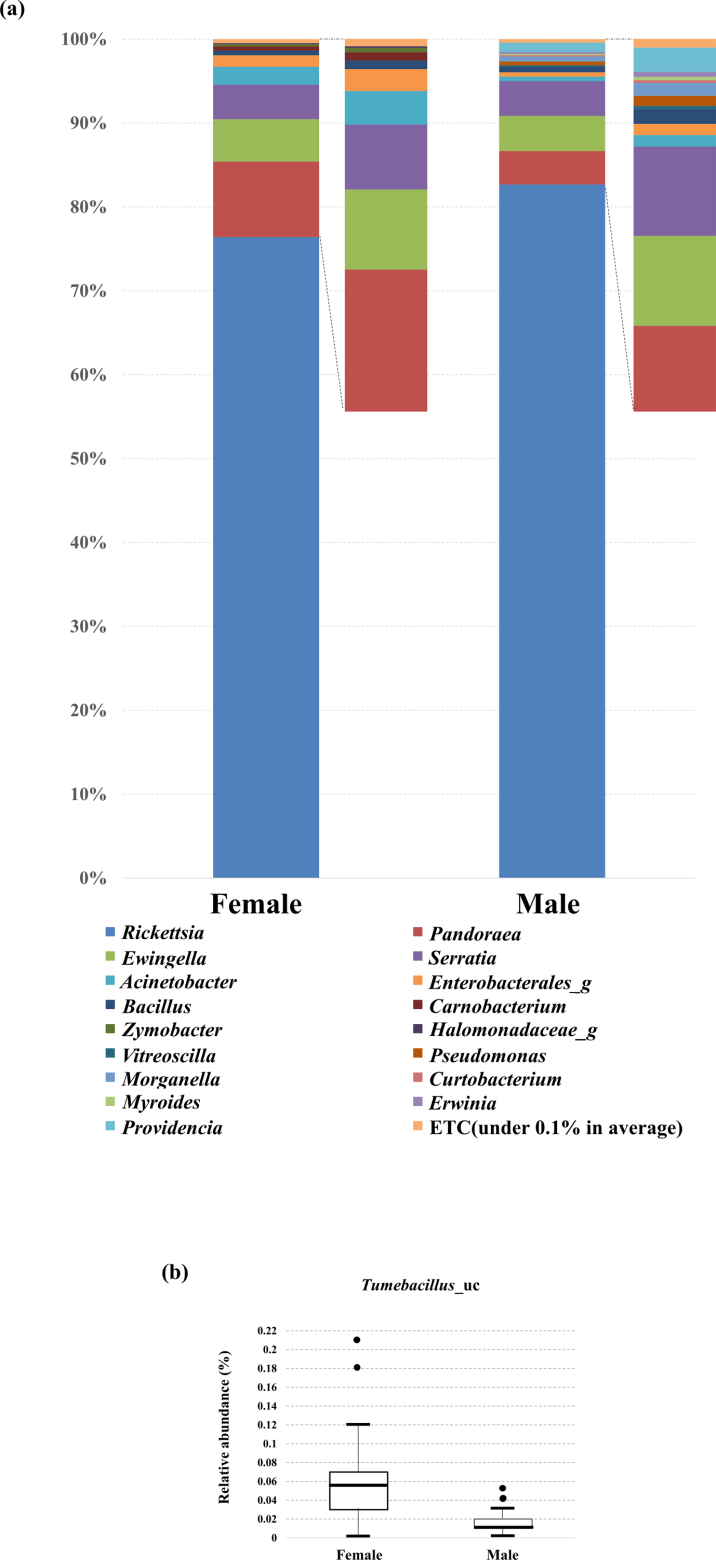
(**a**) The average microbiome composition of female (*n* = 20) and male lovebugs (*n* = 21) at the genus level. Reads accounting for more than 0.1% of total reads are shown. (**b**) Swarm plots of the relative abundance of *Tumebacillus* in female (*n* = 20) and male lovebugs (*n* = 21).

Subsequently, we analyzed the bacterial composition between the two groups and found that *Rickettsia* accounted for 76.43% and 82.52% of the total reads in female and male lovebugs, respectively (Fig. 3a). Differentially abundant bacterial species between groups were identified using linear discriminant analysis effect size (LEfSe). The relative abundance of *Tumebacillus* was higher in the female than in male lovebugs (*P* = 0.00004, Wilcoxon rank-sum test). This pattern was also observed in the relative abundance swarm plots (Fig. 3b). The median relative abundances of *Tumebacillus* in females and males were 0.055 and 0.010, respectively.

### Detection of *Rickettsia* and *Pandoraea* using PCR

**Fig 4:**
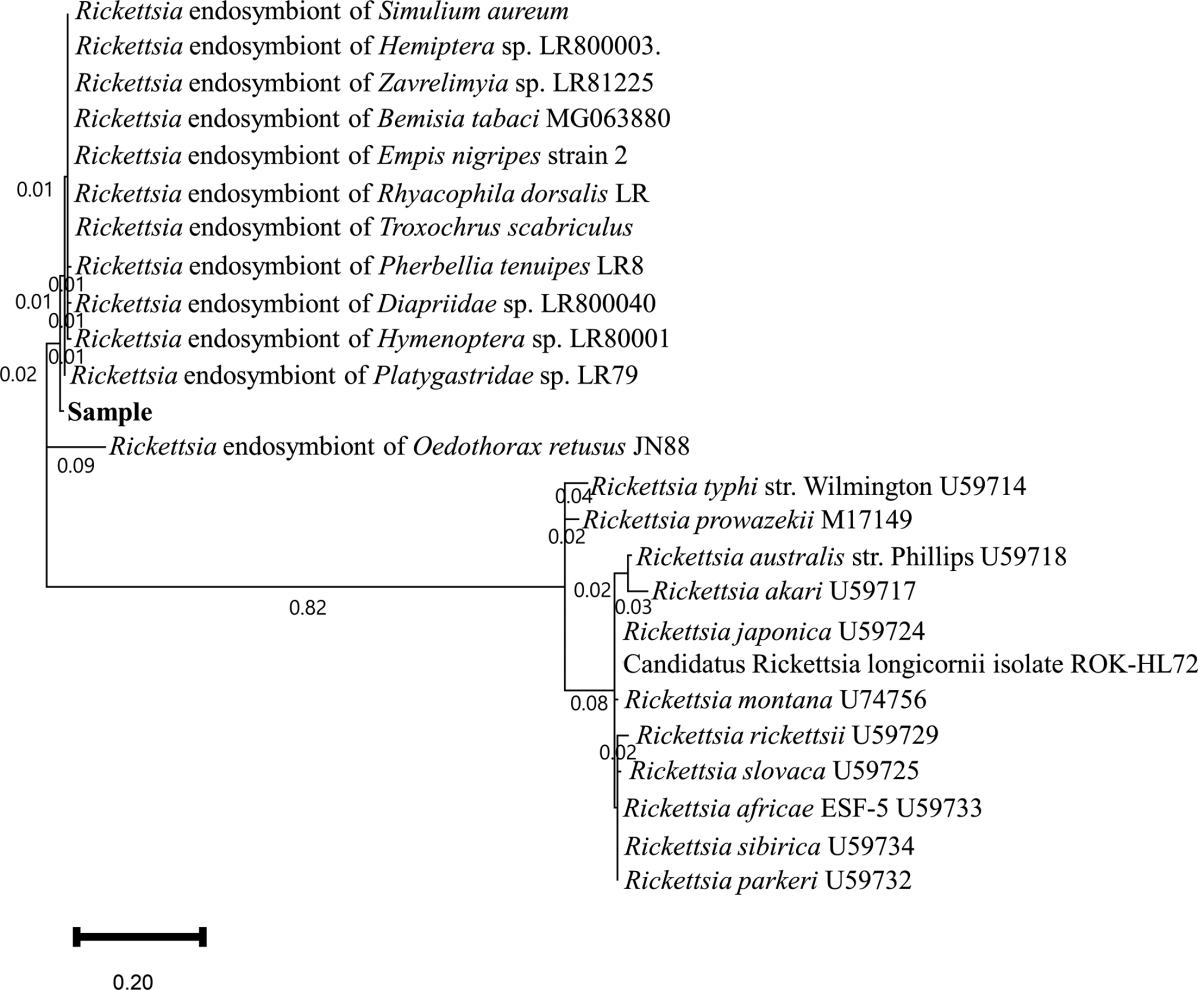
Phylogenetic trees based on partial sequences of *gltA* genes. Sequences of *Rickettsia* sp. detected in the present study were aligned with those retrieved from the GenBank database. The phylogenetic trees were constructed in MEGA 11 software using the maximum likelihood method, employing the Tamura-nei model of nucleotide substitution with 1,000 bootstrap replications.

For samples that tested positive for *Rickettsia* in bacterial metabarcoding using iSeq 100, PCR and Sanger sequencing of the *gltA* gene of *Rickettsia* were performed to identify the strain. The *gltA* gene from the *Rickettsia* of the lovebugs was most similar to that of various arthropod *Rickettsia* endosymbionts, including those from *Platygastridae* sp. (98.94%), *Polydesmus complanatus* (98.41%), *Bemisia tabaci* (98.41%), and *Chrysotus laesus* (98.41%) (Fig. 4). Phylogenetic analysis based on the *gltA* gene sequences revealed that the *gltA* sequence of *Rickettsia* from lovebugs clustered with arthropod *Rickettsia* endosymbionts but did not closely align with spotted fever group rickettsiae (Fig. 4). In addition, while the *Pandoraea* was detected in 11 samples by iSeq 100 analysis, PCR and Sanger sequencing with a *Pandoraea*-specific primer (based on 16S rDNA) revealed 100% sequence similarity to the *Pandoraea oxalativorans* strain DSM 23570 (CP011253.3).

### Full-length 16S rRNA gene sequencing

Additionally, Nanopore long-read sequencing analyzing the full length of 16S rRNA of the four lovebugs showed that *Rickettsia limoniae*, *P. oxalativorans*, *Serratia marcescens*, *Carnobacterium maltaromaticum*, *Acinetobacter apis*, *Enterobacter cloacae*, and so on, were identified (Fig. S2; Table S1). These results were the same as the results obtained using the Illumina platform at the genus level (Fig. 1).

## DISCUSSION

In the summer of 2022, several lovebugs were observed in northwestern Seoul, and in the summer of 2023, this phenomenon also occurred and spread to southern Seoul. Because lovebug outbreaks have been a very rare phenomenon in Korea, their presence has caused fear among residents even if they do not cause immediate health problems in humans (4). Lovebugs, first detected in Florida in 1949, have been observed two times annually at an exceedingly high abundance since the mid-1960s (11, 12). Adult fly swarms were recorded at their peak in 1969, covering nearly one-quarter of Florida, USA (7). In addition, massive lovebug outbreaks can occur in densely populated areas because car exhaust fumes and their components, such as formaldehyde and heptaldehyde, are attractants of *P. nearctica* Hardy (12).

In the current study, PCR showed that the obtained sequence was more similar to *P. longiforceps*, a lovebug from Taiwan, than to species from Florida. According to a previous study conducted in 2022, lovebugs collected from various regions of Korea were identified as either *P. longiforceps* or *P. thulinigra*. Specifically, those collected from the Seoul metropolitan area were identified as *P. longiforceps*, which is consistent with the findings of the present study (4).

Most insect hosts of *Rickettsia* belong to the order Hemiptera, Coleoptera, Diptera, and Hymenoptera (13). They function as primary nutritional symbionts, reproductive manipulators, and insect-vectored plant pathogens in their hosts (14–17). In the present study, *Rickettsia* was detected in all lovebugs, and it exhibited similar nucleotide sequences to those of the *Rickettsia* symbionts of various arthropods and was distant from disease-causing *Rickettsia* sp. (Fig. 4). Additionally, by long-read sequencing (1.5 kb) using Nanopores, we determined that *R. limoniae* was the most abundant bacteria in each of the four lovebugs. (Fig. S2; Table S1). *R. limoniae* is found in the symbionts of non-agricultural insects including the cranefly *Limonia chorea* (18). Additionally, *R. limoniae* is distributed in both the digestive and reproductive systems of the plant-eating bug *Macrolophus pygmaeus* (19, 20). Based on these data, *R. limoniae* in lovebugs are probably symbionts that are expected to play a role in the digestive system.

The second most frequently detected bacterial species in lovebugs was *Pandoraea* sp., which has been isolated from environmental samples such as soil, food, and drinking water (21). Some *Pandoraea* spp. are clinically important and associated with pneumonia, cystic fibrosis, and other underlying chronic lung diseases, as well as with clinical samples such as blood (22, 23). In the present study, the *Pandoraea* sp. in lovebugs had 100% sequence identity with *P. oxalativorans* strain DSM 23570, a known bacterium isolated from soil litter. According to a previous study, *Pandoraea* stably inhabits the intestines of the bean bug *Riptortus pedestris* and partially plays a metabolic role as an intestinal symbiont (24). Therefore, among the bacteria found in lovebugs in this study, *Rickettsia* and *Pandoraea* species are not considered to cause disease.

A limitation of this study is that because the outbreak of lovebugs occurred locally in Seoul, Korea, for a short period (7 days), investigating their microbiome according to climates or habitats was difficult. In the future, more data should be collected by investigating the bacterial composition of lovebugs found in similar climates.

In conclusion, we analyzed the microbiome of lovebugs and identified various bacteria, including *Rickettsia* sp. and *Pandoraea* sp. Importantly, these bacteria were not pathogenic. The detection method employed in the present study can be crucial for rapidly identifying bacterial species in future exotic insect outbreaks that could be harmful to humans.

## MATERIALS AND METHODS

### Sample collection and processing

In June 2023, 41 lovebugs (20 males, 21 females) were collected from the walls of two buildings in Seoul (37.5608377 and 126.9398510) using sterilized conical tubes. Female and male lovebugs were identified based on their morphological characteristics. DNA was extracted from whole-body bugs using a NucleoSpin DNA Insect Kit (Macherey-Nagel, Düren, Germany) according to the manufacturer’s instructions and stored at −20°C until use.

### Illumina sequencing and bioinformatics

The 16S rDNA V4 region was amplified by PCR using 515F (5′-TCG TCG GCA GCG TCA GAT G TGT ATA AGA GAC AGG TGC CAG CMG CCG CGG TAA-3′) and 806R primers (5′-GTC TCG TGGG CTC GGA GAT GTG TAT AAG AGA CAG GGA CTA CHV GGG TWT CTA AT-3′) (25, 26). KAPA HiFi HotStart ReadyMix (Roche Sequencing Solutions, Pleasanton, CA, USA) was used for PCR amplification, which was performed as follows: one cycle at 95°C for 5 min; 25 cycles at 98°C for 30 s, 55°C for 30 s, 72°C for 30 s; and a final cycle at 72°C for 5 min. A limited-cycle (eight cycles) amplification step was performed to add multiplexing indices and Illumina sequencing adapters. Mixed amplicons were pooled, and sequencing was performed using an Illumina iSeq 100 Sequencing System according to the manufacturer’s instructions, utilizing an Illumina iSeq 100 i1 Reagent v2 kit (Illumina, San Diego, CA, USA). Raw read processing started with a quality check and filtering of low-quality (<Q25) reads using Trimmomatic ver. 0.321 (27). After a quality control pass, the sequence data were merged using the fastq_mergepairs command of VSEARCH version 2.13.42, with default parameters (28). The primers were then trimmed using the alignment algorithm of Myers and Miller (29) at a similarity cutoff of 0.8. Nonspecific amplicons that did not encode 16S rRNA were detected using nhmmer (30) in HMMER software ver. 3.2.1 with hmm profiles. Unique reads were extracted, and redundant reads were clustered with unique reads using the derep_full-length command in VSEARCH2. The EzBioCloud 16S rRNA database (31) was used for a taxonomic assignment using the usearch_global command of VSEARCH2, followed by more precise pairwise alignment (29). Chimeric reads were filtered for reads with <97% similarity by reference-based chimeric detection using the UCHIME algorithm (32) and a non-chimeric 16S rRNA database from EzBio-Cloud. After chimeric filtering, reads that were not identified to the species level (<97% similarity) in the EzBioCloud database were compiled, and the cluster_fast command was used to perform *de novo* clustering to generate additional OTUs. Finally, OTUs with single reads (singletons) were omitted from further analysis. All subsequent analyses were performed using EzBioCloud, a commercially available ChunLab bioinformatics cloud platform for microbiome research (http://www.ezbiocloud.net). Reads were normalized to 10,000 to perform the analyses (33). We computed the Shannon index (34) and performed PCoA (35). The Wilcoxon rank-sum test was used to determine differences in the number of OTUs and the Shannon index between the two groups. Significant differences in the relative abundance between the two groups at the phylogenetic level were assessed using LEfSe.

Additionally, the microbiome analysis was performed using the QIIME 2 pipeline v.2022.11.1 (36). Raw reads were demultiplexed and then trimmed using the q2-cutadapt plugin (37). Trimmed sequences were denoised using the DADA2 package (38). The taxonomic classification of the ASVs was performed using the classify-consensus-blast classifier of the q2-feature-classifier plugin (39) against the SILVA v.138.99 database for 16S ASVs.

The methods for the full-length 16S rRNA gene deep sequencing are provided in the supplementary file.

### PCR assay

To identify the lovebug species, PCR was performed using specific primers for the mitochondrial COI sequences used in previous studies (40). The primer sequences were as follows: LCO1490, 5′-GGTCAACAAATCATAAAGATATTGG-3′ (forward) and HCO2198, 5′-TAAACTTCAGGGTGACCAAAAAATCA-3′ (reverse). PCR was performed in an Applied Biosystems Veriti Thermal Cycler (Applied Biosystems, Foster City, CA, USA) with the following conditions: denaturation at 95°C for 3 min, 45 cycles at 95°C for 1 min, 48°C for 1 min, 72°C for 1.5 min, and final extension at 72°C for 3 min.

In addition, the *gltA* gene was PCR amplified to identify the *Rickettsia* species in the lovebugs. The primer sequences were as follows: *gltA*, 5′-GGCTAATGAAGCGGTAATAAATATGCTT-3′ (forward) and 5′-TTTGCGACGGTATACCCATAGC-3′ (reverse) (41). PCR was performed with the following conditions: denaturation at 95°C for 5 min, and then 40 cycles of denaturation at 95°C for 30 s, annealing at 52°C for 1 min, and elongation at 72°C for 30 s (42).

To confirm *Pandoraea* species, PCR was performed using the following primers: 5′-GGGCTYAACCTGGGAACTGCATTC-3′ (forward) and 5′-CGRYTTGGCRRCCCTCTGTACCG-3′ (reverse). After initial denaturation for 2 min at 94°C, 20 amplification cycles were completed, each consisting of 1 min at 94°C, 45 s at 68°C, and 1 min at 72°C. A final extension of 10 min at 72°C was applied (43).

Thereafter, the PCR products were sequenced at Bionics Co. (Seoul, Korea). An NCBI BLAST search was performed to compare the obtained sequences with those available in GenBank (https://www.ncbi.nlm.nih.gov/genbank/).

### Phylogenetic analysis

Alignment and phylogenetic reconstruction were performed using the build function of ETE3 3.1.2 as implemented on GenomeNet (https://www.genome.jp/tools/ete/). Alignment was performed using MAFFT v6.861b with default options (44). The maximum likelihood tree was inferred using IQ-TREE 1.5.5 ran in ModelFinder and tree reconstruction (45). The best-fit model, according to the Bayesian information criterion, was K3Pu + I. Tree branches were tested using SH-like aLRT with 1,000 replicates.

## Data Availability

The authors confirm that the data supporting the findings of this study are available in the article and/or its supplementary material. The metagenomic data of all samples were deposited in the NCBI database under BioProject PRJNA952810.

## SUPPLEMENTAL MATERIAL

Fig. S1 spectrum.03809-23-s0001.tif Microbiome composition of each lovebug at the genus level.

Fig. S2 spectrum.03809-23-s0002.tif Microbiome composition of each lovebug at the species level.

Supplementary methods spectrum.03809-23-s0003.docx Full-length 16S rRNA gene sequencing on Nanopore MinION.

Supplemental material spectrum.03809-23-s0004.docx Legends.

Table S1 spectrum.03809-23-s0005.xlsx Reads of the bacterial microbiome of the four lovebugs at the species level using Nanopore MinION.

ASM does not own the copyrights to Supplemental Material that may be linked to, or accessed through, an article. The authors have granted ASM a non-exclusive, world-wide license to publish the Supplemental Material files. Please contact the corresponding author directly for reuse.
