# Supplementary figures and images for: Microbiome of lovebug (Plecia longiforceps) in Seoul, South Korea
Source: Microbiol Spectr. 2024 May 29;12(7):e03809-23. doi: 10.1128/spectrum.03809-23 (PMC11218492; doi:10.1128/spectrum.03809-23)

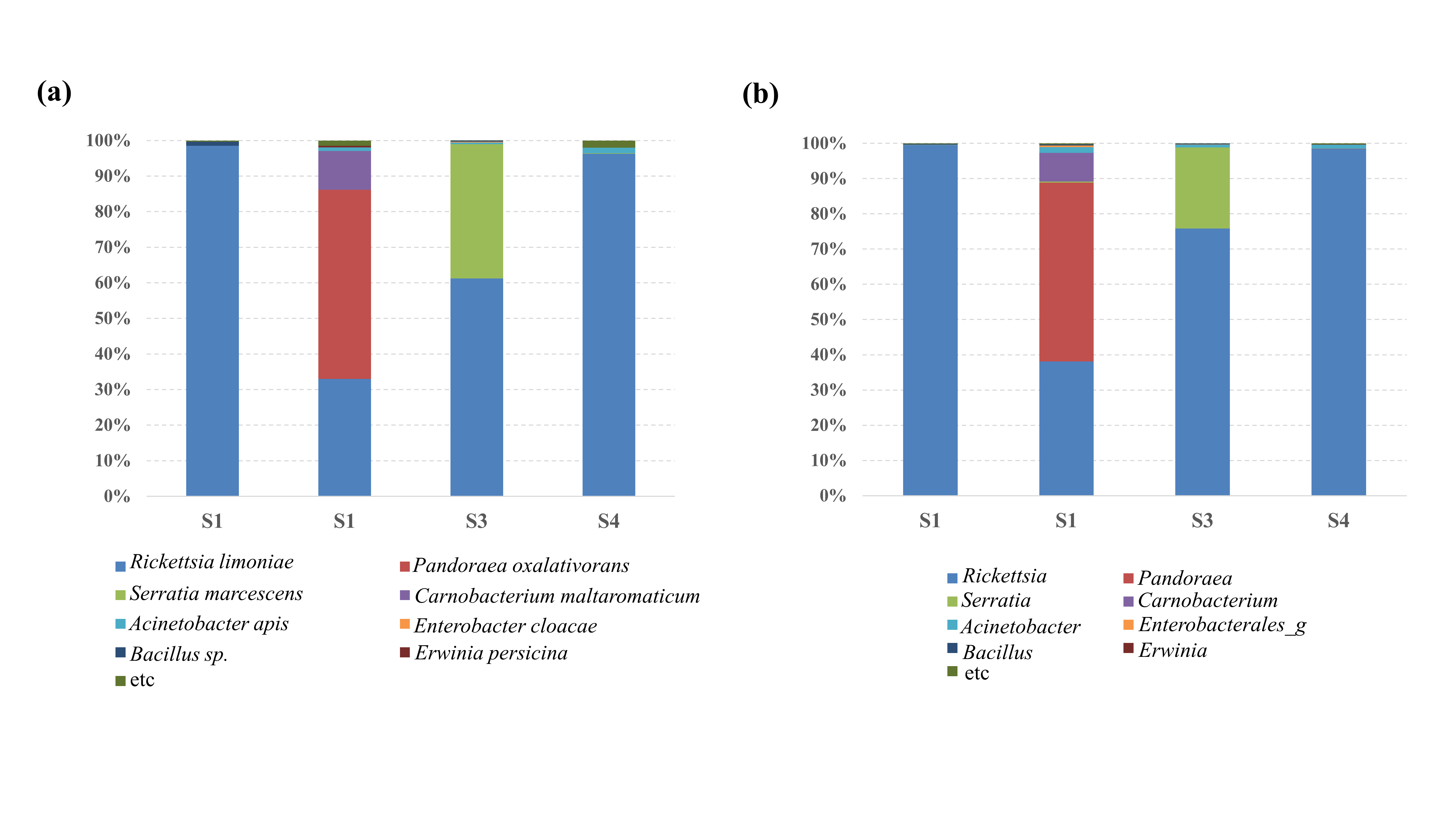

Supplement: Fig. S2 — Microbiome composition of each lovebug at the species level. [file spectrum.03809-23-s0002.tif]
