# Supplementary material for: Microbiome of lovebug (Plecia longiforceps) in Seoul, South Korea
Source: Microbiol Spectr. 2024 May 29;12(7):e03809-23. doi: 10.1128/spectrum.03809-23 (PMC11218492; doi:10.1128/spectrum.03809-23)
Supplement: Supplementary methods — Full-length 16S rRNA gene sequencing on Nanopore MinION. [file spectrum.03809-23-s0003.docx]

**Supplementary methods**

**Full-length 16S rRNA gene sequencing on Nanopore MinION**

Primer PCR with rapid adapter attachment chemistry generated 16S rRNA gene amplicons with 5′ ends for simplified post-PCR adapter attachment following the manufacturer’s instructions. To amplify the V1-V9 region of the 16S rRNA gene, the following inner primers were used with 16S rRNA gene-specific sequences: forward primer (27F) with the anchor sequence 5′-AGA GTT TGA TCM TGG CTC AG-3′ and reverse primer (1492R) 5′-GGT TAC CTT GTT ACG ACT T-3′. The PCR amplification of 16S rRNA genes was performed using Phusion Plus DNA Polymerase (Thermo Scientific™, Wilmington, MA, USA) in a total volume of 25 μL containing inner primer pairs (1 pM each) and the barcoded outer primer mixture (12.5%) from Native Barcoding Kit 24 V14 (SQK-NBD114.24; Oxford Nanopore Technologies, Oxford, UK). Amplification was performed with the following PCR conditions: initial denaturation at 95 °C for 5 min, 25 cycles of 98 °C for 30 s, 55 °C for 30 s, and 72 °C for 30 s, followed by a final extension at 72 °C for 5 min. Amplified DNA was purified using AMPure® XP (Beckman Coulter) and quantified using NanoDrop® 1000 (Thermo Fischer Scientific, Waltham, MA, USA). A total of 20 ng of DNA was incubated with 5 μL of the adapter at room temperature for 20 min. The prepared DNA library (12 μL) was mixed with 37.5 μL of a sequencing buffer and 25.5 μL of loading beads, loaded onto the R10.4.1 flow cell (FLO-MIN114; Oxford Nanopore Technologies), and sequenced on MinION™ Mk1B. MINKNOW software ver. 23.11.2 (Oxford Nanopore Technologies) was used for data acquisition.

The base-calling of the raw fast5 files was performed using Dorado (Version 7.2.13, Oxford Nanopore Technologies Ltd., UK) in fast (fast model, 400 bps) mode. For the bioinformatics analysis, Nanofilt v2.7.1 was used to filter raw reads on quality and read length (De Coster et al., 2018). cutadapt v1.15 (Martin, 2011) was used to trim the extensions and primers, and CD-hit v4.8.1, a program that clusters reads based on short words rather than sequence alignment, was used to cluster the reads based on a set percentage of similarity (W. Li et al., 2002). The clustered reads were subsequently aligned using Minimap2 v2.17 (H. Li, 2018). Based on these alignments, Racon v1.4.13 was used to build the initial draft consensus sequence of each cluster (Vaser, Sović, et al., 2017), which was then polished using Medaka v1.1.2 (Oxford Nanopore Technologies Ltd., UK). We compiled a pipeline, Decona, to automate these processing steps (https://github.com/Saskia-Oosterbroek/decona). The raw base-called reads were first trimmed using a read-length filter at 100–2100 bases for fragments of the 1.5-kb primer pair dataset. A cluster similarity of 80% was selected by carefully checking different clustering percentages (data not shown). Medaka-polished consensus sequences were created from each cluster larger than 25 reads. The commands used to run Decona were as follows: 1.5 kb: decona -f -l 100 -m 2100 -q 10 -c 0.80 -n 25 -k 10 -M.
