# Supplementary material for: Microbiome of lovebug (Plecia longiforceps) in Seoul, South Korea
Source: Microbiol Spectr. 2024 May 29;12(7):e03809-23. doi: 10.1128/spectrum.03809-23 (PMC11218492; doi:10.1128/spectrum.03809-23)
Supplement: Supplemental material — Legends. [file spectrum.03809-23-s0004.docx]

**Fig. S1.** Microbiome composition of each lovebug at the genus level (*n* = 41) using the SILVA database. Reads accounting for more than 0.1% of total reads are shown.

**Fig. S2.** Microbiome composition of each lovebug at the species level (*n* = 4) using (a) Nanopore MinION for full-length 16S rRNA gene comparing with (b) the existing findings performed using the Illumina platform.

**Table S1.** Reads of the bacterial microbiome of the four lovebugs at the species level using Nanopore MinION.

**Supplementary methods**

Full-length 16S rRNA gene sequencing on Nanopore MinION
